# Developing Customized Dental Miniscrew Surgical Template from Thermoplastic Polymer Material Using Image Superimposition, CAD System, and 3D Printing

**DOI:** 10.1155/2017/1906197

**Published:** 2017-02-09

**Authors:** Yu-Tzu Wang, Jian-Hong Yu, Lun-Jou Lo, Pin-Hsin Hsu, CHun-Li Lin

**Affiliations:** ^1^Department of Biomedical Engineering, National Yang-Ming University, Taipei, Taiwan; ^2^School of Dentistry, College of Medicine, China Medical University, Taichung, Taiwan; ^3^Plastic & Reconstructive Surgery, Department of Surgery, Chang Gung Memorial Hospital, Taiwan

## Abstract

This study integrates cone-beam computed tomography (CBCT)/laser scan image superposition, computer-aided design (CAD), and 3D printing (3DP) to develop a technology for producing customized dental (orthodontic) miniscrew surgical templates using polymer material. Maxillary bone solid models with the bone and teeth reconstructed using CBCT images and teeth and mucosa outer profile acquired using laser scanning were superimposed to allow miniscrew visual insertion planning and permit surgical template fabrication. The customized surgical template CAD model was fabricated offset based on the teeth/mucosa/bracket contour profiles in the superimposition model and exported to duplicate the plastic template using the 3DP technique and polymer material. An anterior retraction and intrusion clinical test for the maxillary canines/incisors showed that two miniscrews were placed safely and did not produce inflammation or other discomfort symptoms one week after surgery. The fitness between the mucosa and template indicated that the average gap sizes were found smaller than 0.5 mm and confirmed that the surgical template presented good holding power and well-fitting adaption. This study addressed integrating CBCT and laser scan image superposition; CAD and 3DP techniques can be applied to fabricate an accurate customized surgical template for dental orthodontic miniscrews.

## 1. Introduction

The orthodontic miniscrew provides skeletal anchorage with the advantages of being relatively inexpensive, easily implemented, and predictable enough to be used routinely in medical practice [1]. Nonetheless, the failure rates of interradicularly inserted miniscrews are considered still too high [2]. The miniscrew placement poses a challenge to the orthodontist because of the limited space available for miniscrew placement and the potential risk for root damage, puncture to the maxillary sinus, and neurovascular damage during miniscrew placement procedures [3]. Safe and optimal miniscrew stabilization requires ideal placement point and trajectory. Several methods have been developed to achieve precise and safe miniscrew placement in interradicular sites; however, they cannot guarantee precise placement [4–11]. A controllable method for miniscrew placement and direction is important for orthodontists.

Traditionally, 2-dimensional (2D) information in the radiographs is usually used for surgical sites planning to minimize the root damage risks [7–9]. A metal wire-guide is used to superimpose radiograph images for analyzing surgical plan coordinates, distances, and angles and the corresponding miniscrew assessment [7]. However, metal wire bending skills are necessary and difficult to bend in shallow sulcus areas [5, 6]. Otherwise, direction of 2D radiograph should be parallel to the occlusal guide which limited 2D projection images and still cannot solve the 3-dimensional spatial error occurring during miniscrew placement or eliminate the risk for root injury [5, 6]. Custom-made surgical guides and templates have recently been proposed for transferring computed tomography (CT) images to the surgical site and outlining the ideal miniscrew axis, to promote safe miniscrew placement into the dentoalveolar bone [4, 10, 11]. However, fabricating the accuracy and fit of individualized surgical guides is time-consuming and requires extensive advance preparation because CT images only isolate the hard tissue positions (jawbone or teeth). Another template needed to be fabricated on the stone cast with the vacuum-formed technique for providing radiopaque landmarks for further CT scan alignment. Complex double CT scan, landmark definition, and image processing procedures are needed to identify the accurate position and thickness of the soft tissue (mucosa) that comes in direct contact with the surgical templates [1, 4, 7].

This study integrates image superposition of CBCT/laser scanning, computer-aided design (CAD), and rapid prototyping technologies to develop a simplified, accurate technique for producing customized miniscrew surgical templates with adaptive fitness between the mucosa and template for accurate miniscrew insertion paths.

## 2. Materials and Methods

### 2.1. Image Superimposition and Miniscrew Inserted Path Definition

**Figure 1:**
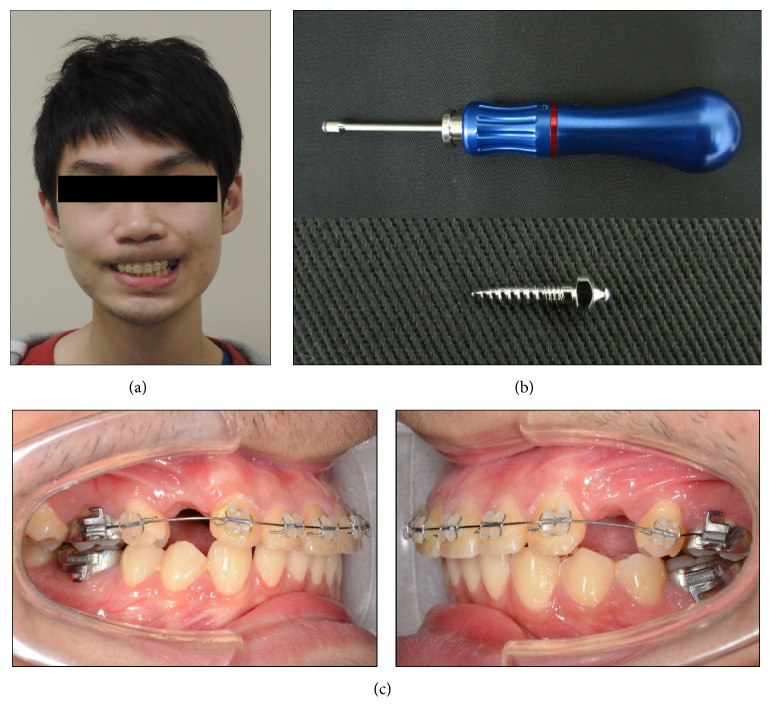
(a) A 25-year-old male patient with skeletal Class II occlusal features; (b) dual-thread miniscrew (materials: Ti6Al4V, Bomei Co, Ltd, Taoyuan, Taiwan) 1.6 mm in diameter and 8 mm in length was selected as the anchor implants; (c) two miniscrews were planned for insertion at the canine-second premolar space of the left/right buccal sites to provide a controlled anterior retraction and intrusion with power chain for the maxillary canines/incisors.

The patient was a 25-year-old male patient with skeletal Class II occlusal features (Figure 1(a)). Two dual-thread miniscrews (materials: Ti6Al4V, Bomei Co, Ltd, Taoyuan, Taiwan) 1.6 mm in diameter and 8 mm in length were selected as the anchor implants and planned for insertion at the canine-second premolar space of the left/right buccal sites to provide a controlled anterior retraction and intrusion with power chain for the maxillary canines/incisors (Figures 1(b) and 1(c)).

**Figure 2:**
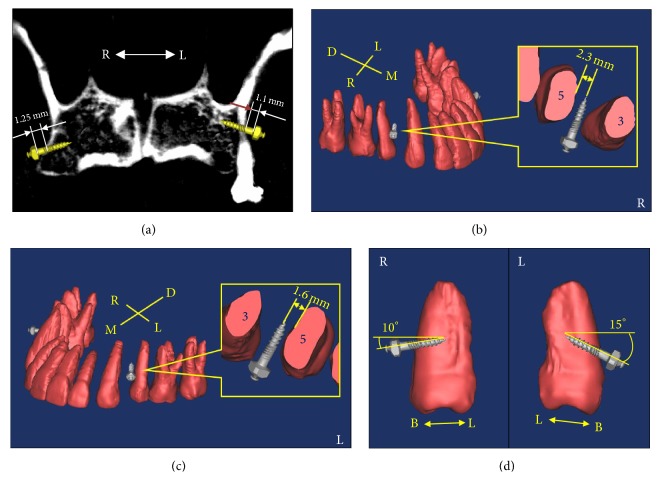
(a) Section image of CBCT reconstructed model to identify the contours of cortical, cancellous bone and teeth for planning the miniscrews microthreads to contact the cortical bone layer; (b) and (c) 3D solid model of the maxillary bone with teeth was reconstructed for surgical miniscrew planning at right and left sites; (d) two miniscrews were planned to insert to the occlusal surface inclination at angles of 15° and 10° for the left and right placement sites.

CBCT (Cone-Beam Computed Tomography, Asahi AZ3000, Kyoto-shi, Japan) scan with 0.155 mm interval was performed on the patient to reconstruct the 3D maxillae model. All DICOM CT cross-section image data were processed on a personal PC using commercially available image processing software (Amira, v4.1, Mercury Computer Systems, Chelmsford, MA). This approach allowed identifying the contours of different hard tissues (cortical, cancellous bone and teeth) and those contours were extracted and converted into mathematical entities. A 3D solid model of the maxillary bone with teeth was reconstructed (Figures 2(a)–2(c)).

**Figure 3:**
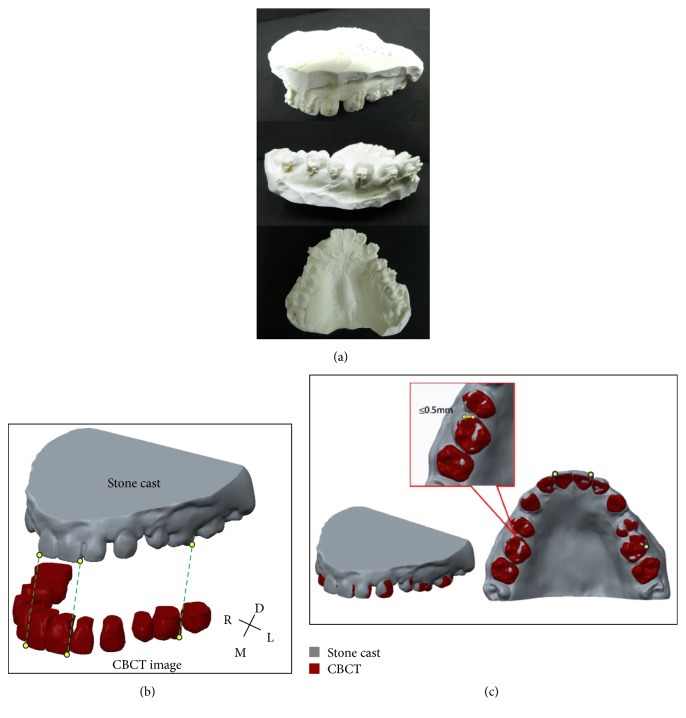
(a) Stone cast was fabricated from the patient (including teeth/mucosa/bracket contour profiles); (b) reconstructed models from CBCT and laser scanning images; (c) solid models of maxillary bone with teeth reconstructed using CBCT images and maxillary dental arches with mucosa acquired using laser scanning were superimposed using the common register positions at the distoincisal angle of left/right incisors and distobuccal cusp of left/right first molar.

In order to ensure that the surgical template would fit well with the patient's teeth and soft tissue (mucosa), a maxilla impression was taken to make a stone cast (Figure 3(a)). The stone cast was then scanned using a 3D surface laser scan system (3Shape Scanners, 3D Scan CO., Ltd., USA) to make a digital 3D impression model for recording the geometry and profile of the teeth and mucosa (Figure 3(a)). Solid models of maxillary bone with teeth reconstructed using CBCT images and maxillary dental arches with mucosa acquired using laser scanning were superimposed using the common register positions at the distoincisal angle of left/right incisors and distobuccal cusp of left/right first molar (Figures 3(b) and 3(c)). The superimposition model consisted of hard tissue (bone and teeth) positions to allow miniscrew visual insertion planning accurate orientation and teeth/mucosa/bracket contour profiles to permit surgical template fabrication with well-fitting adaption.

The CAD miniscrew models were imported and placed in safe and optimal positions, that is, between the canine and second premolar space and as close as possible to premolar for canine distal drive when the teeth roots were visualized (Figures 2(b) and 2(c)). The distance between the second premolar and canine roots for the miniscrew were measured. The periodontal ligament is about 0.15 to 0.38 mm thick on average (assumed as 0.4 mm in this study) [4, 12, 13] and the miniscrew diameter in this study was 1.6 mm. The safe distance between the second premolar root and miniscrew axis was calculated and should be larger than 1.2 mm, that is, the sum of the periodontal ligament thickness is 0.4 mm and miniscrew radius is 0.8 mm for safe placement. Two miniscrews were inserted to the occlusal surface inclination at angles of 15° and 10° for the left and right placement sites (Figure 2(d)). The insertion depth was controlled to allow the miniscrew microthreads to contact the cortical bone layer for better stabilization (Figure 2(a)).

### 2.2. Surgical Template Fabrication and Interfacial Adaption Test

**Figure 4:**
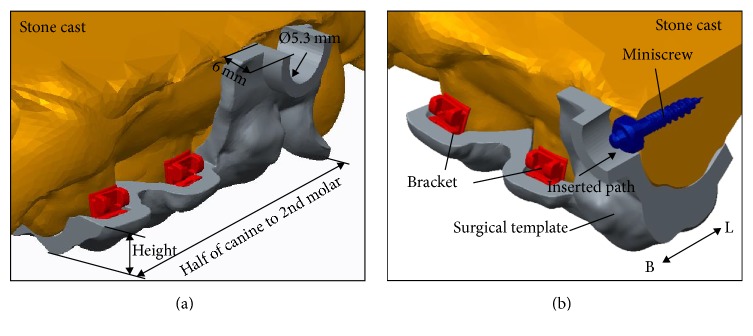
(a) and (b) The customized surgical template was 1.8 mm thick layer average offset based on the teeth/mucosa/bracket contour profiles and ranged from half canine to second molar. The surgical template height was designed to cover the entire occlusal surface and extend to half the crown height to protect the orthodontic brackets bonded onto each tooth. The miniscrew drill paths and guided cylinders were designed 5.3 mm in diameter and 6 mm in height.

**Figure 5:**
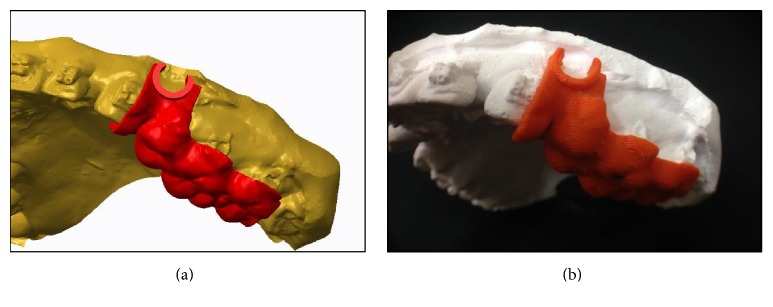
(a) The assembly solid models of stone cast of dental arch and left site surgical template; (b) the surgical template (left site) was fabricated using a 3DP printer with 0.254 mm slicing additive manufacturing and wore on the stone cast.

The customized surgical template CAD model was fabricated with a 1.8 mm thick layer average offset based on the teeth/mucosa/bracket contour profiles in previous superimposition models, ranging from half canine to second molar (Figures 4(a) and 4(b)). The surgical template height was designed to cover the entire occlusal surface and extend to half the crown height to protect the orthodontic brackets bonded onto each tooth. The miniscrew drill paths and guided cylinders (5.3 mm in diameter and 6 mm in height) were generated in the surgical template CAD model according to previous 3D information from the visual surgical plan (Figures 4(a) and 4(b)). The solid surgical template model can be exported as a stereo-lithographic (STL) file that can be loaded into a fused deposition modeling (FDM) 3D printing (3DP) printer with 0.254 mm slicing additive manufacturing (Dimension 1200es SST, Strayasys, Ltd., Minnesota, USA) to duplicate the acrylonitrile butadiene styrene (ABS) (ABS-P430, Strayasys, Ltd., Minnesota, USA) polymer material template (Figure 5). The ABS material is a thermoplastic polymer with mechanical properties suitable to endure impact resistance and toughness.

**Figure 6:**
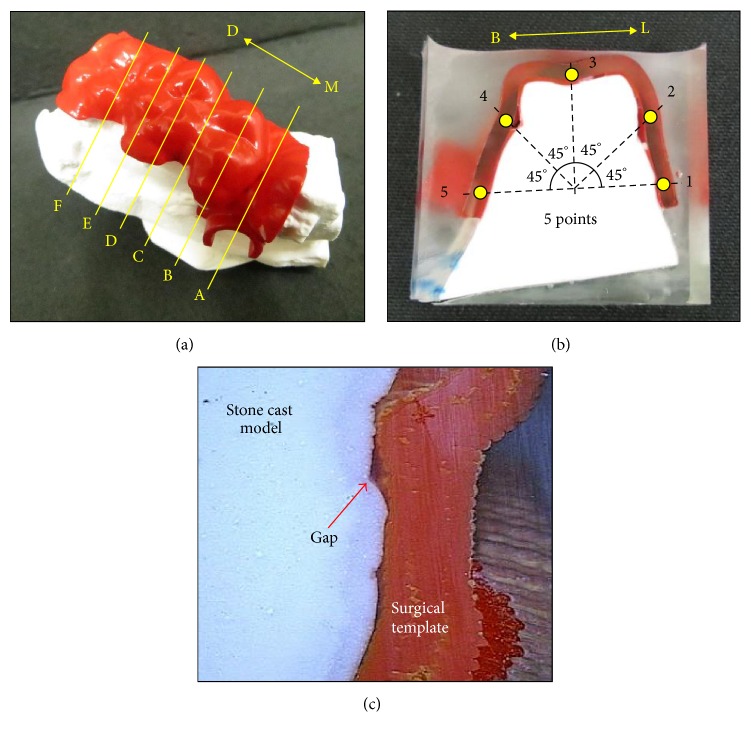
(a) Customized surgical template was fitted on the teeth/bracket/mucosa stone cast model of the patient to perform the interfacial adaption test. Six (A–F) buccal-palatal direction sections were obtained using a diamond saw; (b) and (c) each cutting section was scanned using a noncontact video measurement system to measure the gap sizes for 5 points with 45° separation in the counterclockwise direction on each section.

In order to evaluate the interfacial fitness accuracy, an interfacial adaption test was performed to measure the gap sizes between the surgical template and teeth/mucosa tissue [1]. The customized surgical template was fitted onto teeth/mucosa stone cast models and embedded into clear rectangular test boxes with epoxy resin (Truetime Industrial Co., Taiwan) to provide a stable base. The resin block was sectioned in the buccal-palatal direction from the second premolar to molar with 6 section slices using a low speed diamond saw with copious cooling (CL50 Precision Saw, Top Tech Machines Co., Ltd., Taiwan) (Figure 6). The section slices were scanned using a noncontact video measurement system (SVP-2010, ARCS Co., Ltd., Taichung, Taiwan) to measure the gap sizes at 5 points with 45° separation in the counterclockwise direction on each section.

### 2.3. Clinical Test

**Figure 7:**
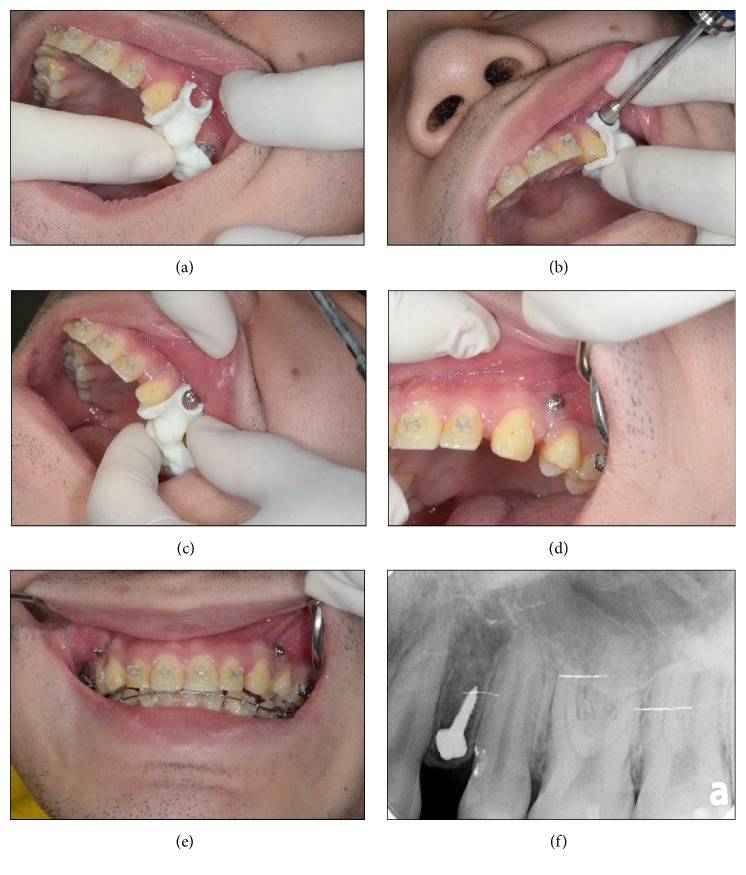
(a) The surgical templates were placed intraorally onto the teeth by a clinician; (b) to (e) showed the clinical application of the customized surgical template; (f) X-rays were taken after implantation to assess whether damage to the peripheral tissue occurred for assessing miniscrew stability.

The surgical templates were placed intraorally onto the teeth by a clinician after the patient was given a local anesthetic (Figure 7(a)). The fitting adaption accuracy between the surgical template and teeth should be confirmed again with the surgical template held in place using the patient's bite force. Once the template was seated the patient was asked to bite the template to expose the guide surgical sites. The miniscrews were then placed with a screwdriver (Figures 7(b)–7(e)) until the screw head bottom slightly touched the mucosa. X-rays were taken after implantation to assess whether damage to the peripheral tissue occurred for assessing miniscrew stability (Figure 7(f)). The patient was given instructions for postoperative care and antibiotics were prescribed (500 mg amoxicillin 4 times daily for 3 days).

## 3. Results and Discussion

Safe distances between the second premolar root and miniscrew axis were designed 1.6 mm and 2.3 mm for the left and right buccal insertion placements, respectively (Figures 2(b) and 2(c)). The clinical application indicated that two miniscrews were placed smoothly, safely, and without problems. No inflammation, screw loosening, or other symptoms of discomfort had occurred one week after surgery.

Miniscrews are always placed at an inclined angle, obviously limiting 2D image measurement. CT scan therefore must be used to measure the interradicular spaces for accurate and reproducible results. CT scan has become an important diagnostic tool for the craniofacial region and is used for many applications including the study of treatment planning for orthognathic and reconstructive surgery, bone grafting, distraction osteogenesis, and dental implantology. However, the CT characteristic only presents/identifies deeper anatomical hard tissue, which often limits its applicability in surgery guiding templates [4, 14, 15]. Much effort and numerous methods such as double CT scans for hard tissue and the radiographic template and landmark definition used with metal wires/balls were proposed to identify the contour profile and thickness of soft tissue (mucosa) that comes into direct contact with surgical templates [1, 4, 7]. This study proposes an image superimposition method using laser scan and CBCT images to improve the surgical template interfacial fitting adaption problem. Laser scanning can be used as a complementary tool for designing surgical templates because the images obtained from laser scanning can present the patient's dental arch with a good degree of similarity and may enhance the holding power and stability of the surgical template.

The stability and inherent support of the surgical template is a crucial factor. The template in this study was supported using 3 surfaces—occlusal teeth and buccal/palatal mucosa. The bite forces on the template provided support to keep it stable. The results of the interfacial adaption test showed that the average gap sizes in the different tooth sections were found to be smaller than 0.5 mm (total average 0.30 ± 0.10 mm) (Tables 1 and 2) and confirmed that the image superimposed method can produce a surgical template with good holding power and well-fitting adaption. It is difficult to compare other template holding power accuracy directly to that of our fitting adaption which was not evaluated in these literatures [4–11]. Nevertheless, surgical template with well-fitting adaption dominates and is relative to the precise miniscrew placement. The study by Liu et al. showed that the linear distomesial deviation of placing miniscrews was 0.42 mm at the tip. This deviation may be useful to compare the accuracy of our surgical template fitting adaption (average 0.30 mm in gap size) and confirmed that the image superimposed method can produce a surgical template with good holding power and well-fitting adaption.

The 3D superposition digital model from CBCT and laser scanning images produced accurate hard tissue relationship positions (bone and teeth) to simulate miniscrews placed in the safe position with ideal inclined angle and positions. Dual-thread miniscrews with correct microthread pitch (parametrical relationship with macrothread pitch) in the cortical bone region can improve primary stability and enhance mechanical retention [16]. The microthread portion in the miniscrews must be precisely controlled to contact the cortical bone layer in the visual planning procedure, that is, total lengths of microthread and smooth (sliding) portions below the screw head (3 mm) need to be larger than the cortical bone layer and soft tissue (mucosa) total thickness. In our clinical test case, that is, canine distal drive, the cortical bone layers/mucosa thicknesses at the left and right buccal sites were 1.15 mm/1.1 mm and 1.53 mm/1.25 mm, respectively (Figure 2(a)). This case indicated that the miniscrew microthread can contact the cortical bone layer when the miniscrew head bottom is controlled to slightly touch the mucosa during insertion.

3DP in medical applications has been widely used in several broad categories, including the creation of customized surgical templates, implants, and anatomical models because of its medical product customization and personalization, cost-effectiveness, and design and manufacturing democratization. However, the FDM 3DP printer with 0.254 mm slicing additive manufacturing was used in this study. The 3DP mechanic must take into account the high resolution to meet the well-fitting template accuracy requirement. Otherwise, biocompatibility considerations, especially for the toxicity of 3DP material, must be tested before template fabrication. The patient may delay the overall orthodontic treatment time because the timing of producing the surgical template was about one week from reconstructing the patient image to complete the 3DP fabrication. Time-consuming surgical template production must be compressed when the surgical template is required for miniscrew placement.

A new method for integrating CBCT/laser scan image superimposition, CAD system, and 3DP techniques was developed in this study and applied to fabricate an accurate customized surgical template for orthodontic miniscrews. More clinical applications can be applied to verify the feasibility of the proposed method. For clinical consideration, this customized surgical template can be used for situations when molar intrusion/extrusion, molar uprighting, molar distalization, buccoversion/scissors bite, and molar mesial drive are to be performed. It is not applicable when there is no sufficient room for interradicular miniscrew placement or when extremely low maxillary sinus floor is observed. In addition, severely unstable occlusal surfaces of the anchor teeth can also be excluded when a surgical template is to be used.

## Figures and Tables

**Table 1:** Gap size of the interfacial adaption test of left surgical template (unit: mm).

Section	Placement	Point					Average	SD
		1	2	3	4	5		
A	Canine	0.29	0.40	0.11	0.09	0.13	0.20	0.12
B	2nd Premolar	0.33	0.06	0.30	0.05	0.09	0.17	0.12
C	2nd Premolar/1st Molar	0.55	0.27	0.35	0.54	0.47	0.44	0.11
D	1st Molar	0.33	0.20	0.32	0.48	0.40	0.35	0.09
E	1st Molar	0.53	0.38	0.17	0.10	0.28	0.29	0.15
F	2nd Molar	0.38	0.67	0.29	0.16	0.25	0.35	0.18
Total average							0.30 ± 0.10	

**Table 2:** Gap size of the interfacial adaption test of right surgical template (unit: mm).

Section	Placement	Point					Average	SD
		1	2	3	4	5		
A	Canine	0.45	0.47	0.83	0.25	0.44	0.49	0.19
B	2nd Premolar	0.17	0.41	0.13	0.15	0.54	0.28	0.17
C	2nd Premolar/1st Molar	0.21	0.41	0.07	0.44	0.31	0.29	0.14
D	1st Molar	0.08	0.14	0.32	0.20	0.39	0.23	0.11
E	1st Molar	0.07	0.27	0.41	0.30	0.15	0.24	0.12
F	2nd Molar	0.21	0.20	0.05	0.46	0.43	0.27	0.15
Total average							0.30 ± 0.10	
